# Inhibition of mammalian S6 kinase by resveratrol suppresses autophagy

**DOI:** 10.18632/aging.100056

**Published:** 2009-06-03

**Authors:** Sean M. Armour, Joseph A. Baur, Sherry N. Hsieh, Abigail Land-Bracha, Sheila M. Thomas, David A. Sinclair

**Affiliations:** ^1^ Department of Pathology and Paul F. Glenn Laboratories for the Biological Mechanisms of Aging, Harvard Medical School, Boston, MA 02115, USA; ^2^ Department of Medicine, Harvard Medical School and Beth Israel Deaconess Medical Center, Boston, MA 02215, USA; ^3^ These authors contributed equally to this work

**Keywords:** Resveratrol, autophagy, p70 S6 kinase, S6K1, autophagosome, nutrient withdrawal

## Abstract

Resveratrol is a plant-derived polyphenol that promotes health and disease resistance in rodent models, and extends lifespan in lower organisms. A major challenge is to understand the biological processes and molecular pathways by which resveratrol induces these beneficial effects. Autophagy is a critical process by which cells turn over damaged components and maintain bioenergetic requirements. Disruption of the normal balance between pro- and anti-autophagic signals is linked to cancer, liver disease, and neurodegenerative disorders. Here we show that resveratrol attenuates autophagy in response to nutrient limitation or rapamycin in multiple cell lines through a pathway independent of a known target, SIRT1. In a large-scalein vitro kinase screen we identified p70 S6 kinase (S6K1) as a target of resveratrol. Blocking S6K1 activity by expression of a dominant-negative mutant or RNA interference is sufficient to disrupt autophagy to a similar extent as resveratrol. Furthermore, co-administration of resveratrol with S6K1 knockdown does not produce an additive effect. These data indicate that S6K1 is important for the full induction of autophagy in mammals and raise the possibility that some of the beneficial effects of resveratrol are due to modulation of S6K1 activity.

## Introduction

Autophagy is an essential process by which eukaryotic cells turn over long-lived cytosolic components, clear damaged proteins and organelles, and maintain bio-energetic requirements during conditions of nutrient and growth factor withdrawal [1]. Degradation and recycling of cellular components can involve the uptake of small amounts of cytoplasm at the vacuole or lysosome surface (microautophagy) or, in response to a strong stimulus such as starvation, the formation of specialized double membraned-organelles termed autophagosomes, which engulf larger portions of cytoplasm or organelles before fusing with a vacuole or lysosome (macroautophagy), hereafter referred to simply as autophagy [1-3]. While this process is an important part of the normal balance between anabolic and catabolic processes and can prolong survival during nutrient limitation, autophagy is also an alternate death pathway that facilitates type II programmed cell death [4-6]. For this reason, imbalances in this pathway can contribute to seemingly diverse pathologies.

Resveratrol is a small polyphenol that extends the lifespan of simple model organisms, ostensibly by mimicking caloric restriction [7,8]. In rodents, resveratrol protects from a variety of age-related diseases including cancer, cardiovascular disease, neurodegeneration, obesity and diabetes [9-14]. Although there is evidence that some of resveratrol's actions are mediated by activation of the SIRT1 deacetylase, the mechanisms underlying the numerous beneficial effects of resveratrol remain to be elucidated.

It has previously been reported that 24-48 hours of resveratrol treatment induces autophagy in cancer cells grown in rich media, suggesting a mechanism by which resveratrol might enhance cell death and suppress tumor growth [15-18]. Here we report the effect of resveratrol treatment on the normal induction of autophagy over 4-6 hours following nutrient withdrawal in tumor and non-tumor cell lines. In contrast to the activation of the autophagic pathway observed in tumor cells in complete media, we find that resveratrol markedly inhibits the starvation-induced autophagic response. We show that this effect does not require SIRT1, and identify p70 S6 kinase (S6K1) as a target of resveratrol that is responsible for the inhibition of starvation-induced autophagy.

**Figure 1.**
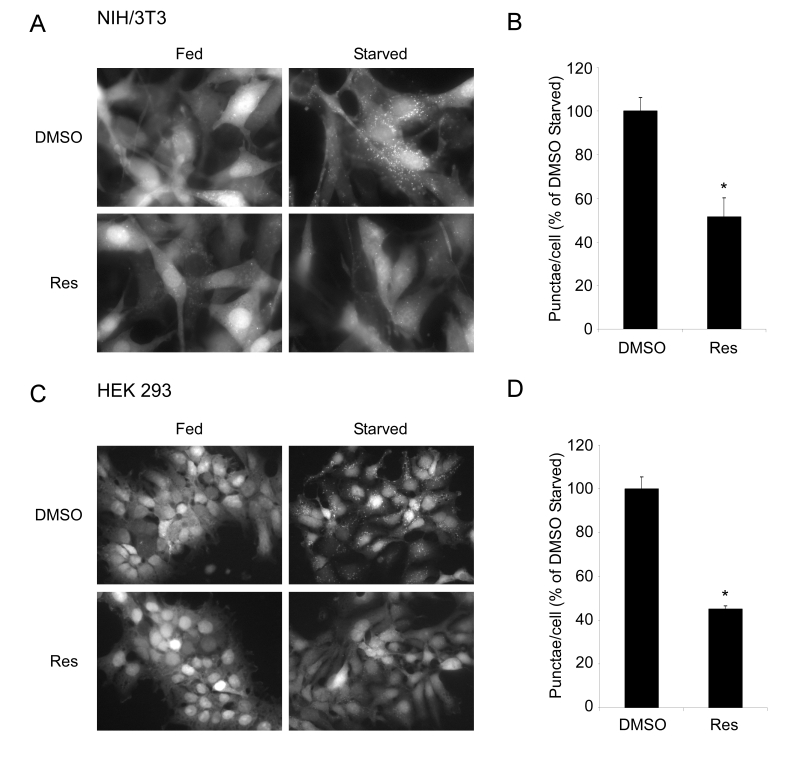
Resveratrol inhibits autophagy in mammalian cells. (**A**) NIH/3T3 cells stably expressing the GFP-LC3 fusion protein were subjected to nutrient withdrawal by replacing growth media (Fed) with Earle's buffered saline solution (Starved) and treated with either DMSO or 50 μM resveratrol (Res) for 2 hours. Representative fields at 63X (oil immersion) magnification are shown. (**B**) Quantification of punctae/cell in (**A**) of at least 4 fields per treatment are represented as a percentage of the starved DMSO treated cells. (**C**) HEK293 cells stably expressing the GFP-LC3 fusion protein were subjected to starvation and either DMSO or 50 μM Res for 6 hours. Representative fields at 40X magnification are shown. (**D**) Quantification was performed on HEK293 cells as in (**B**). Error bars represent s.e.m. * (p < 0.0022).

## Results

### Suppression of nutrient starvation- and rapamycin-induced autophagy by resveratrol

Autophagy is an important component of the cellular response to nutrient stress and growth factor withdrawal. We therefore tested whether resveratrol treatment would influence regulation of autophagy under these conditions. Autophagy was assessed by monitoring the relocalization of a component of the autophagy machinery, LC3, from the cytoplasm to the forming autophagosome [19]. NIH/3T3 cells or HEK293 cells stably expressing a GFP-LC3 fusion protein were generated and were induced to undergo autophagy in the presence or absence of resveratrol. Treatment with resveratrol resulted in a dramatic reduction in the number of starvation-induced GFP-LC3 punctae (Figure 1). Similar inhibitory effects on autophagy were observed in other cell lines, including human tumor cell lines (HeLa and U2OS), as well as mouse embryonic fibroblasts (MEFs) using monodansylcadaverine (MDC), a fluorescent compound that stains late autophagosomes (Supplementary Figures 1 and 3) [20]. Since previous studies have described an increase in autophagy following 24 hours of resveratrol treatment in nutrient rich media, we also tested the effects of resveratrol under these conditions. Consistent with these results, we observed an induction of autophagosome formation in cells treated with resveratrol for 24 hours in complete media containing serum (Supplementary Figure 2). Thus, the influence of resveratrol on autophagy is context dependent, and in the case of autophagy induced by nutrient limitation, resveratrol is inhibitory.

**Figure 2.**
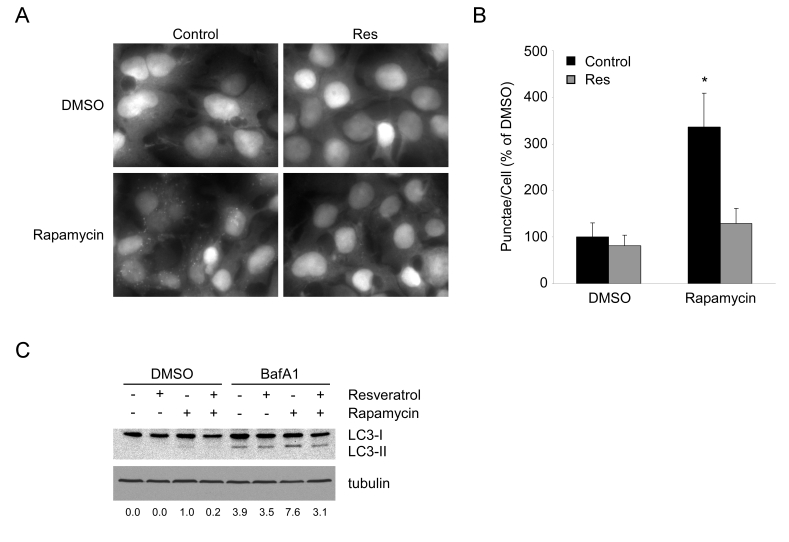
Resveratrol suppresses autophagy under TOR inhibition. (**A**) HEK293 cells stably expressing GFP-LC3 growing in complete media were pretreated with DMSO or 50 μM resveratrol (Res) for 1 hour, prior to addition of DMSO or 200 nM rapamycin for 4 hours. 40X magnification fields have been cropped and zoomed for ease of punctae visualization. (**B**) Quantification of punctae/cell from (**A**) of 10 fields per treatment are represented as a percentage of DMSO treated cells. Error bars represent s.d.m. * (p < 0.0001) (**C**) HEK293 GFP-LC3 cells were pretreated for 1 hour with DMSO or resveratrol and subsequently treated with DMSO or 1 mM rapamycin in the presence or absence of 100 nM Bafilomycin A1 for 4 hours. A representative western blot of endogenous LC3 and tubulin are shown. Numbers represent the ratio of LC3-II to tubulin for each condition normalized to Rapamycin in the absence of BafA1.

Rapamycin is an inhibitor of the nutrient sensing mTOR-Raptor complex and has been shown to induce autophagy [21,22]. In yeast, it had been shown that resveratrol can reverse some markers of autophagy induced by rapamycin [23]. Consistent with these results and similar to the results seen under nutrient limitation, rapamycin-induced autophagy is almost completely abrogated by resveratrol treatment (Figure 2A and B). Decreased GFP-LC3 punctae could be due either to increased flux or a block in autophagy. To distinguish between these two possibilities, we examined LC3-II accumulation with and without Bafilomycin A1, an inhibitor of lysosome degradation. Under both conditions, resveratrol was able to block the accumulation of LC3-II, indicating a suppression of autophagy rather than an enhancement of lysosomal clearance (Figure 2C).

**Figure 3.**
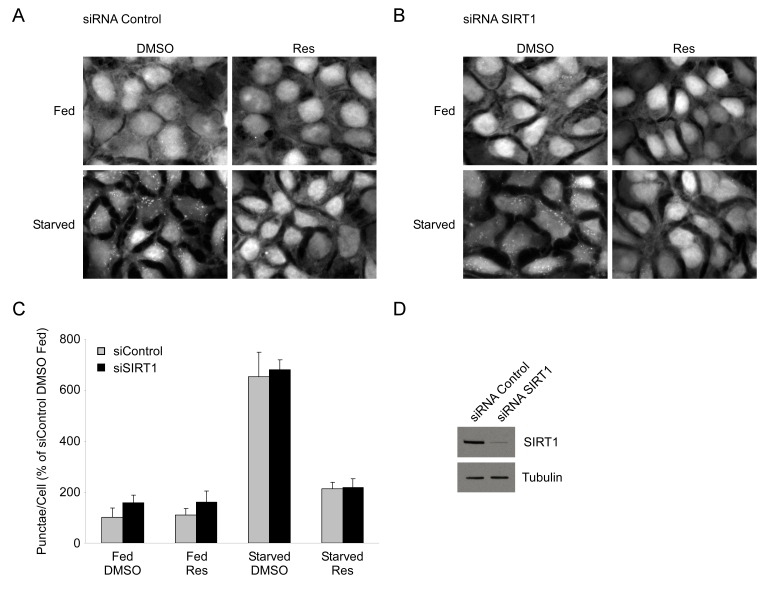
Resveratrol suppresses autophagy independently of SIRT1. HEK293 cells stably expressing GFP-LC3 were transfected with either a control siRNA (**A**) or an siRNA directed against SIRT1 (**B**) for 72 hours. Subsequently, cells were subjected to nutrient starvation with or without 50 μM resveratrol (Res) treatment for 4 hours. 40X magnification fields have been cropped and zoomed for ease of punctae visualization. (**C**) Quantification of punctae/cell from (**A**) and (**B**) of 4 fields are represented as a percentage of fed DMSO treated control siRNA cells. Error bars represent s.d.m. (**D**) Representative western blot showing typical knockdown of SIRT1 by siRNA transfection in HEK293 GFP-LC3 cells.

We next tested whether SIRT1, an NAD^+^-dependent deacetylase that is activated by resveratrol [8], was required for this effect. To test this hypothesis, HEK 293 GFP-LC3 cells were transfected with either control siRNA or an siRNA directed against the SIRT1 deacetylase and subsequently nutrient starved in the presence or absence of resveratrol. We found that both control and SIRT1 knockdown cells displayed a similar level of induction of GFP-LC3 punctae and that resveratrol still produced an equivalent suppression of autophagy (Figure 3). Consistent with these results, SIRT1+/+ and SIRT1-/- MEFs induced autophagy in response to nutrient withdrawal, and in both cell lines the inhibitory effect of resveratrol on autophagy was comparable (Supplementary Figure 3). These data indicate that inhibition of starvation-induced autophagy by resveratrol is molecularly distinct from the induction seen in previous studies and is not mediated by the SIRT1-dependent pathway that has previously been described [24]. It will be interesting to explore the differences in these systems that engage or disengage SIRT1 during autophagic induction.

### Kinase profiling of resveratrol *in vitro*

**Figure 4.**
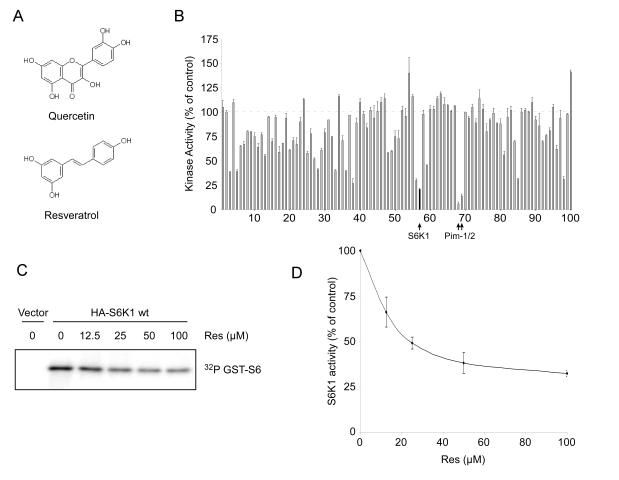
Resveratrol inhibits S6K1 *in vitro*. (**A**) Structural similarity between resveratrol and quercetin, a known kinase inhibitor. (**B**) Kinase inhibition profile for resveratrol at 20 μM obtained using KinaseProfiler™ (Upstate). Dashed line represents 100% activity as compared to control. Black filled-in bar on the graph indicates S6K1. Complete data set is provided in Supplementary Table 1. Error bars represent s.d.m. (**C**) Phosphorylation of recombinant GST-tagged S6 by immunoprecipitated HA-S6K1 under increasing concentrations of resveratrol (Res). Autoradiograph depicts S6K1 phosphorylation of GST-S6. (**D**) Average of three separate kinase assay experiments as performed in (**C**). Densitometry was performed using NIH ImageJ. Error bars represent s.e.m.

Resveratrol has previously been shown to inhibit several kinases including PKC and Src [25] and is structurally similar to the flavanoid quercetin (Figure 4A), which is an inhibitor of PI 3-kinase [26]. Therefore, we hypothesized that the effect of resveratrol on autophagy could be related to inhibition of one or more upstream kinases. To test this, we performed an *in vitro *kinase screen and determined an inhibition profile for resveratrol. Out of 100 kinases tested, Jak2, NLK, p70 S6 kinase (S6K1), Pim-1, and Pim-2 emerged as potential targets of resveratrol (Figure 4B, Supplementary Table 1). With the exception of S6K1, these kinases play primary roles in the hematopoietic system, and were thus viewed as unlikely to have been responsible for the effect we were studying. Further-more, although JNK had previously been shown to play a positive role in autophagy, its activity was not significantly inhibited by resveratrol at this dose [27]. On the other hand, S6K1 is known to play a requisite role in the regulation of autophagy in *Drosophila *[28], making it a promising candidate for the *in vivo *target of resveratrol that is responsible for inhibition of autophagy.

To confirm the inhibition of S6 kinase by resveratrol *in vitro*, we determined the effect of the compound on immunoprecipitated HA-tagged S6K1 from HEK293 cells using purified GST-tagged full-length recombinant S6 ribosomal protein (S6) as a substrate. In agreement with the primary screen, we found that resveratrol inhibited the activity of S6 kinase in a dose-dependent manner, exhibiting an IC50 of ~25 μM (Figure 4C and D, and Supplementary Figure 4).

**Figure 5.**
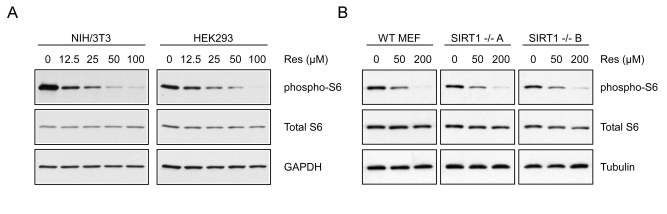
Resveratrol inhibits S6K1 in intact cells. (**A**) NIH/3T3 or HEK293 cells were treated for 30 minutes with increasing doses of resveratrol and whole cell extracts were western blotted for the indicated proteins. (**B**) WT or two separate lines of SIRT1-/- MEFs (**A** and **B**) were treated with increasing doses of resveratrol for 30 minutes and analyzed by western blot.

### Resveratrol-mediated inhibition of S6 kinase activity *in vivo*

To test whether resveratrol alters S6 kinase activity in cells, we treated NIH/3T3 and HEK293 cells with resveratrol and analyzed the phosphorylation status of S6, a well-characterized downstream target of S6 kinase. After 30 minutes, phosphorylation of S6 was dramatically decreased in a dose-dependent manner in both cell lines at concentrations consistent with the IC50 determined *in vitro *(Figure 5A). These results were observed in a variety of different mammalian cell lines and were independent of SIRT1, since treatment of either SIRT1-/- or wildtype MEFs with resveratrol resulted in a similar decrease in S6 phosphorylation (Figure 5B). It is interesting to note that the doses required to inhibit phosphorylation of S6 were significantly higher in MEFs than those in other lines. Consistent with this observation, inhibition of autophagy in MEFs also required a higher concentration than in other cell lines (Supplementary Figure 3).

### Regulation of starvation-induced autophagy by S6 kinase 1

**Figure 6.**
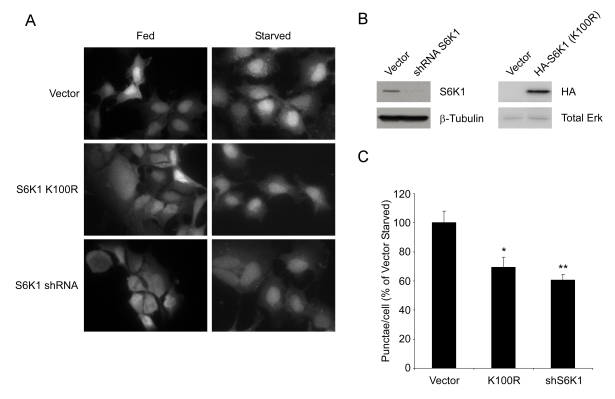
S6K1 is required for autophagy in mammalian cells. (**A**) HEK293 cells stably expressing GFP-LC3 were infected with retrovirus encoding a dominant negative S6K1 (K100R) or with lentivirus encoding a specific shRNA directed against human S6K1 and subjected to nutrient withdrawal by replacing supplemented media (Fed) with EBSS for 4 hours (Starved). Representative fields at 63X (oil immersion) magnification are shown. (**B**) Efficiency of S6K1 knockdown and expression of HA-tagged S6K1 (K100R) in HEK293 GFP-LC3 cells. (**C**) Quantification of punctae/cell from (**A**) of at least 9 fields per treatment are represented as a percentage of the starved vector control cells. Error bars represent s.e.m. * (p < 0.0015) ** (p < 0.0002)

To test whether inhibition of S6K1 might account for suppression of autophagy by resveratrol, GFP-LC3 expressing HEK293 cells were infected with retrovirus encoding a dominant-negative (K100R) mutant of S6 kinase 1 or lentivirus encoding an shRNA against human S6K1. Cells expressing K100R S6K1 or knocked down for S6K1 expression showed a significant reduction in the number of GFP-LC3 punctae compared to control cells following nutrient withdrawal (Figure 6). These results demonstrate that S6 kinase inhibition is sufficient to suppress the induction of autophagy under nutrient-starved conditions. Similar observations have been made previously in *Drosophila *[28], but had not been extended to mammalian systems. Mieulet et al. have observed that a normal basal level of autophagy still proceeds in S6K1;S6K2-/- muscle cells [29]; however, it is not clear whether nutrient deprivation or rapamycin can induce an increase in autophagy in this system. Moreover, studies in these mice suggest that mitogen signaling through p90rsk might compensate for the loss of S6 kinase signaling [30]. Our results support the view that under normal circumstances S6K1 plays a role in the induction of autophagy in response to nutrient deprivation.

### S6 kinase dependence of resveratrol-mediated suppression of autophagy

**Figure 7.**
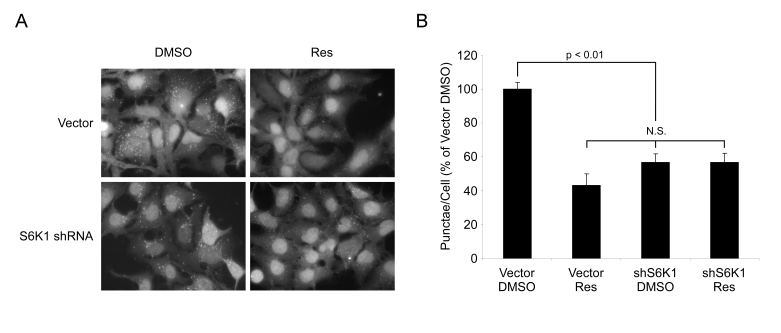
Resveratrol does not affect autophagy in the absence of S6K1. (**A**) HEK293 GFPLC3 cells were infected with shRNA S6K1 lentivirus or control virus and treated with EBSS (Starved) for 4 hrs ± 50 μM resveratrol (Res). Representative fields at 63X (oil immersion) magnification are shown. (**B**) Quantification of punctae/cell from (**A**) of at least 4 fields per treatment are represented as a percentage of DMSO treated starved vector control cells. Error bars represent s.e.m. N.S. = not significant.

To provide additional evidence that the effects of resveratrol are mediated via S6K1, HEK293 cells infected with control virus or a virus encoding shRNA against S6K1 were treated with resveratrol and subjected to nutrient withdrawal. Individually, S6K1 shRNA and resveratrol both dramatically reduced the number of GFP-LC3 punctae, and resveratrol treatment in the absence of S6K1 produced no further decrease (Figure 7A). Quantification of punctae revealed no statistically significant difference between any of the experimental treatments, all of which were significantly different when compared to control cells (Figure 7B). These data indicate that S6 kinase is required for the full induction of autophagy in response to nutrient withdrawal in mammals, and lend further support to the view that the reduced level of autophagy in resveratrol-treated cells is due to inhibition of S6K1.

## Discussion

Resveratrol has been characterized as an activator of SIRT1/Sir2, which are members of a family of enzymes that promote longevity in lower organisms [7,8,31-33]. In rodents, resveratrol has many beneficial effects including cancer prevention, cardio- and neuroprotective effects, and improvements in insulin sensitivity, although the extent to which these effects are mediated by SIRT1 is not yet clear [9-14]. Here we identify a novel target of resveratrol activity, S6K1, that may have important implications for understanding the mechanisms by which resveratrol increases health in both lower organisms and mammals*. *We suggest that inhibition of S6K1 may act in parallel to or in concert with activation of SIRT1 to modulate lifespan and health.

A previous study described an important positive role for SIRT1 in autophagy; however, we did not observe a noticeable change in the rate of autophagy when altering SIRT1 activity in various assays. It was recently shown that FK866, a NAMPT inhibitor, leads to reduced NAD^+^and is sufficient to induce autophagy. This suggests that SIRT1 was also not required to induce autophagy, since NAD^+^is required for SIRT1 activity [34]. It will be important to understand the differences in these studies in order to clarify under what conditions SIRT1 can regulate autophagy.

The role of S6K in autophagy has generated considerable confusion due to seemingly conflicting data. Inhibition of the upstream kinase, mTOR, induces autophagy [3] and accordingly, ribosomal protein S6 phosphorylation has been inversely correlated with autophagy [35]. In addition, ATG1 has been shown to inhibit S6 kinase activity by blocking its ability to be phosphorylated on Thr 389 [36]. However, in *Drosophila*, S6 kinase is required for induction of autophagy in response to starvation or genetic manipulation of the insulin-signaling pathway [28]. These data suggest that under some conditions, mTOR and S6K can oppose each other or that S6K may be activated in an mTOR independent manner. Consistent with the work on *Drosophila* S6K, our data support a positive role for S6K1 in autophagic induction in mammals, and raise important questions about how this could occur. It is reasonable to envisage the existence of a novel substrate of mammalian S6 kinase that is required for initiation or maturation of autophagic vesicles, which is targeted only in the absence of mTOR activity, and that this target's phosphorylation may be required for autophagy to proceed. This mechanism would provide a dual switch for the initiation of autophagy, facilitating tighter control of a process that has both positive and negative implications for the cell.

It is interesting to consider why resveratrol treatment might have an inhibitory effect on starvation-induced autophagy, yet stimulate the inappropriate induction of autophagy in nutrient rich media. One possibility is that insulin signaling is the key difference. Under nutrient withdrawal, where insulin signaling is minimal, inhibition of S6K1, leading to a reduction in autophagy, might be the dominant effect of resveratrol. On the other hand, when autophagy is held in check by robust signaling through insulin-PI3K-Akt-mTOR (fed conditions), disrupting this pathway might lead to the induction of autophagy over time. It will be interesting to test the effects of resveratrol on autophagy in animals, especially under starvation or tumor models, where we might observe a similar duality of function.

Negative regulation of homologs of S6 kinase in lower organisms promotes beneficial effects on health and lifespan. In yeast, deletion of Sch9, the homolog of mammalian S6K/Akt, protects against age-dependent defects in a yeast model of aging and cancer and extends chronological lifespan [37-39]. In *Drosophila *it has previously been shown that expression of a dominant negative S6K can extend lifespan [40] and results in increased resistance to oxidative stress [41]. Resveratrol's effects on lifespan and resistance to oxidative stress are well established; therefore, it would be exciting if some of these effects are due to suppression of S6K activity.

Of considerable interest is the fact that resveratrol-treated mice exhibit most of the phenotypes of S6K1-/- mice when fed a high fat diet [12,13,42]. In comparison to control animals, both resveratrol treated and S6K1-/- mice have significantly less body fat and their peripheral tissues remain highly sensitive to insulin. Moreover, mitochondrial number and activity are increased in both S6K1-/- and resveratrol-treated mice. It is therefore interesting to speculate that resveratrol's ability to modulate S6K1 activity might be responsible for at least some of the therapeutic effects observed in recent studies.

A major unanswered question is how resveratrol can mediate protection from a diverse range of disease processes such as cancer, neurodegenerative disease, and liver disease. One possible explanation proposed by Howitz and Sinclair to explain these effects is the Xenohormesis Hypothesis [8,43]. The theory proposes that organisms have adapted to sense stress-induced molecules produced by other species in their environment, and use these cues to induce a protective response in preparation for adversity. This may explain why many stress-induced phytochemicals, such as resveratrol, quercetin, and pterostilbene are beneficial for health and seem to act through multiple pathways [9]. In the case of resveratrol, this includes SIRT1 activation, inhibition of cyclooxygenase and NFκB, induction of antioxidant enzymes, and activation of AMPK *in vivo *[13,44], in addition to the inhibition of the leukemia-related kinases and S6K1 reported here (Supplementary Table 1).

Taken together, these results suggest that the requirement for S6 kinase in autophagy is evolutionarily conserved from flies to mammals. Our observation that S6K1 is necessary to achieve full induction of autophagy in response to nutrient withdrawal opens the door to future studies to discover downstream targets of S6K1 that may be important regulators of autophagy. Furthermore, we establish S6 kinase as a novel target for resveratrol action which may play an important role in mediating its beneficial effects on disease processes and aging in diverse organisms.

## Methods

Cell lines, lysates, and antibodies. HEK293, HEK293T, U2OS, HeLa, NIH/3T3, and mouse embryonic fibroblast cells were maintained in DMEM (GIBCO, Carlsbad, CA) +10% FBS (Gemini Bio-Products, West Sacramento, CA) + 100 units/ml penicillin/100 μg/ml streptomycin (GIBCO, Carlsbad, CA) +2mM glutamine (GIBCO, Carlsbad, CA) at 37 ºC +5% CO2. SIRT1+/+ and SIRT1-/- MEFs were a gift from K. Chua, R. Mostoslavsky, and F. Alt (Harvard Medical School, Boston, MA). HEK293 cells stably expressing GFP-LC3 were maintained in media supplemented with 2 mg/ml puromycin (InvivoGen, San Diego, CA). For S6 phosphorylation experiments, cells were grown overnight and were subjected to various doses of resveratrol (LALILAB Inc, Durham, NC) or DMSO control for the indicated times. Cells were subsequently washed once in PBS and lysed in ice cold PBS +0.5% NP-40 (Sigma Aldrich, St. Louis, MO) + Complete Mini EDTA-free protease inhibitor tablet (Roche, Basel, Switzerland) + 1:100 phosphatase inhibitor cocktail 1 (Sigma Aldrich, St. Louis, MO). Following normalization of protein by Bradford assay (Bio-Rad, Hercules, CA), samples were resolved by SDS-PAGE and western blotted with the indicated antibodies. Antibodies for p70 S6 kinase, Phospho-S6 ribosomal protein (Ser240/244), S6 ribosomal protein, and total Erk antibodies were obtained from Cell Signaling Technology (Beverley, MA). Polyclonal HA antibody was obtained from Sigma Aldrich (St. Louis, MO). Monoclonal β-tubulin antibody was obtained from Upstate (Lake Placid, NY). Monoclonal antibody for GAPDH was obtained from Abcam (Cambridge, MA). Anti-LC3 peptide-based polyclonal antibody was a gift from J. Brugge (Harvard Medical School, Boston, MA).

Plasmids, RNAi, and virus infection. The plasmids pBABE HA-S6K1 K100R, pRK7 HA-S6K1 wt, pRK7 HA-S6K1 K100R, and pGEX-GST-S6 were gifts from J. Blenis (Harvard Medical School, Boston, MA). Lentiviral based shRNAs directed against S6K1 were obtained from B. Hahn and The RNAi Consortium (Harvard Medical School/Broad Institute). pBABE-GFP-LC3 was a gift from J. Debnath (Harvard Medical School, Boston, MA). RISC-free control siRNA and SIRT1 specific siGENOME siRNA (D-003540-05) (Dharmacon, Chicago, IL) were transfected with Lipofectamine RNAiMAX (Invitrogen, Carlsbad, CA) according to manufacturer's specifications. The shRNA sequence used for human S6K1 was (AGCACAGCAA ATCCTCAGACA). Retrovirus and lentivirus were generated by transient transfection of HEK293T cells with packaging plasmids and the target plasmid using polyethyleneimine (PEI). Virus was harvested 48 and 72 hours post-transfection. For disruption of S6K1 function in GFP-LC3 expressing HEK293 cells, cells were infected with retrovirus encoding S6K1 K100R or lentivirus encoding an shRNA directed against S6K1. Cells were assayed for autophagy and protein expression three days after infection.

Autophagy assays. For autophagy studies, GFP-LC3 expressing cells were plated overnight on coverslips. In the case of HEK293 cells glass coverslips were precoated with a solution of 0.1 mg/ml poly-ornithine (Sigma Aldrich, St. Louis, MO) to aid in attachment. Cells were washed twice with PBS and placed in either growth medium or starvation medium, Earle's Balanced Salt Solution (Sigma Aldrich, St. Louis, MO) for the indicated times. In resveratrol experiments, cells were pretreated for 1 hour with either DMSO or resveratrol and then placed in starvation media or treated with 200 nM rapamycin (Calbiochem, San Diego, CA) ± resveratrol (where indicated). The dose of resveratrol used to assay autophagy was 50 μM unless otherwise indicated. Following treatment cells were fixed in 3.7% paraformaldehyde (Sigma Aldrich, St. Louis, MO) and mounted with GEL/MOUNT (Biomedia corp., Foster City, CA). Cells were then visualized on a Zeiss Axiovert with a 63X oil immersion lens and digital photomicrographs were captured with a CCD camera. To quantify GFP-LC3 punctae, at least 4 random fields were imaged and the average number of punctae/cell was calculated. Data sets were compared using Student's t- Test (two-tailed assuming equal variance). For autophagosome staining of SIRT1-/- or SIRT1+/+ MEFs, U2OS, or HeLa, the cells were treated as described for GFP-LC3 samples and then subjected to 30 μM monodansylcadaverine (Sigma Aldrich, St. Louis, MO) in the media for 10 minutes at 37ºC +5% CO2 and then fixed with 3.7% paraformaldehyde and visualized as described. For the LC3 flux assay, HEK293 GFP-LC3 cells pretreated with 50 μM resveratrol for 1 hour followed by treatment with either DMSO or 1 mM rapamycin (Calbiochem, San Diego, CA) in the presence or absence of 100 nM bafilomycin A1 (Sigma Aldrich, St. Louis, MO). Cells were washed once in 4ºC PBS and immediately lysed in 2X Lammeli sample dye and then boiled for 10 minutes. Samples we subsequently run on a 15% polyacrylamide gel and western blotted for LC3 and tubulin. LC3-II/tubulin ratios we quantified with ImageJ (NIH).

Kinase profile and kinase assay. A Kinase profile against 100 kinases listed for resveratrol at 20 μM was generated utilizing Upstate's KinaseProfiler™ service. Assay protocols for each kinase are available from Upstate (Lake Placid, NY). *In vitro *kinase assay was performed as described previously [45]. Briefly, HEK293 cells were transfected with HA-S6K1 wt, HA-S6K1 K100R, or vector as indicated. Cells were treated with 1 μg/ml insulin for 10 minutes prior to harvesting in PBS + 0.5% NP-40 + protease and phosphatase inhibitors. 125 μg of lysate per sample was immunoprecipitated using 0.5 μg/sample of anti-HA High-Affinity (Roche, Basel Switzerland). Beads from immunoprecipitations were washed three times in lysis buffer and once in kinase buffer, and kinase assays were performed with recombinant GST-S6 as substrate (1 μg per assay). In the indicated lanes varying doses of resveratrol or DMSO control were added to the reaction prior to addition of the GST-S6 substrate. All samples were subjected to SDS-PAGE, and 32P incorporation was quantified by using a Bio-Rad (Hercules, CA) Phosphor-Imager and subsequent analysis with ImageJ (NIH).

## Supplementary table

Supplementary Table 1

## Supplementary figures

Supplementary Figure 1 Resveratrol inhibits nutrient-starvation induced autophagy in multiple cell lines. (**A**) MDC staining of U2OS cells subjected to nutrient limitation (Starved) ± 50 μM resveratrol (Res) for 4 hours. (**B**) MDC staining of HeLa cells subjected to nutrient limitation (Starved) ± 50 μM resveratrol (Res) for 4 hours. An expansion of the area in the white box in the far right panels is displayed for clarity.

Supplementary Figure 2 Long-term resveratrol treatment induces autophagy in rich media. (**A**) HEK293 GFP-LC3 expressing cells incubated in complete media plus serum were subjected to 50 or 100 μM resveratrol for 24 hours. (**B**) HEK293 GFP-LC3 cells were treated with EBSS (Starved) ±50 μM resveratrol (Res) for punctae comparison.

Supplementary Figure 3 Resveratrol mediated inhibition of autophagy is independent of SIRT1. (**A**) MDC staining of wild-type (SIRT1+/+) MEFs subjected to nutrient limitation (Starved) ± 200 μM resveratrol (Res) for 4 hours. (**B**) MDC staining of SIRT1-/- MEFs subjected to EBSS (Starved) ± 200 μM Res for 4 hours.

Supplementary Figure 4 Immunoprecipitated S6K1 phosphory-lates GST-S6 *in vitro.* HEK293 cells transfected with vector control (vec), kinase dead (K100R), or wild-type (WT) S6K1, were immunoprecipitated for HA-tagged S6K1, which was subsequently used to phosphorylate full-length GST-S6 ribosomal protein. Top panel is an HA western blot (WB). Middle panel is an autoradiogram indicating phosphorylated GST-S6. The bottom panel is a coomassie stained gel indicating the total GST-S6 in each lane. The black line indicates where the gel was cropped to include only the positive and negative controls for simplicity.

## References

[1] Levine B, Klionsky DJ (2004). Development by self-digestion: molecular mechanisms and biological functions of autophagy. Dev Cell.

[2] Reggiori F, Klionsky DJ (2002). Autophagy in the eukaryotic cell. Eukaryot Cell.

[3] Lum JJ, DeBerardinis RJ, Thompson CB (2005). Autophagy in metazoans: cell survival in the land of plenty. Nat Rev Mol Cell Biol.

[4] Tsujimoto Y, Shimizu S (2005). Another way to die: autophagic programmed cell death. Cell Death Differ.

[5] Shintani T, Klionsky DJ (2004). Autophagy in health and disease: a double-edged sword. Science.

[6] Rubinsztein DC, Gestwicki JE, Murphy LO, Klionsky DJ (2007). Potential therapeutic applications of autophagy. Nat Rev Drug Discov.

[7] Wood JG, Rogina B, Lavu S, Howitz K, Helfand SL, Tatar M, Sinclair D (2004). Sirtuin activators mimic caloric restriction and delay ageing in metazoans. Nature.

[8] Howitz KT, Bitterman KJ, Cohen HY, Lamming DW, Lavu S, Wood JG, Zipkin RE, Chung P, Kisielewski A, Zhang LL, Scherer B, Sinclair DA (2003). Small molecule activators of sirtuins extend Saccharomyces cerevisiae lifespan. Nature.

[9] Baur JA, Sinclair DA (2006). Therapeutic potential of resveratrol: the in vivo evidence. Nat Rev Drug Discov.

[10] Anekonda TS (2006). Resveratrol--a boon for treating Alzheimer's disease. Brain Res Rev.

[11] Jang M, Cai L, Udeani GO, Slowing KV, Thomas CF, Beecher CW, Fong HH, Farnsworth NR, Kinghorn AD, Mehta RG, Moon RC, Pezzuto JM (1997). Cancer chemopreventive activity of resveratrol, a natural product derived from grapes. Science.

[12] Lagouge M (2006). Resveratrol improves mitochondrial function and protects against metabolic disease by activating SIRT1 and PGC-1alpha. Cell.

[13] Baur JA (2006). Resveratrol improves health and survival of mice on a high-calorie diet. Nature.

[14] Parker JA, Arango M, Abderrahmane S, Lambert E, Tourette C, Catoire H, Neri C (2005). Resveratrol rescues mutant polyglutamine cytotoxicity in nematode and mammalian neurons. Nat Genet.

[15] Kueck A, Opipari AW Jr, Griffith KA, Tan L, Choi M, Huang J, Wahl H, Liu JR (2007). Resveratrol inhibits glucose metabolism in human ovarian cancer cells. Gynecol Oncol.

[16] Ohshiro K, Rayala SK, Kondo S, Gaur A, Vadlamudi RK, El-Naggar AK, Kumar R (2007). Identifying the estrogen receptor coactivator PELP1 in autophagosomes. Cancer Res.

[17] Ohshiro K, Rayala SK, El-Naggar AK, Kumar R (2008). Delivery of cytoplasmic proteins to autophagosomes. Autophagy.

[18] Opipari AW Jr, Tan L, Boitano AE, Sorenson DR, Aurora A, Liu JR (2004). Resveratrol induced autophagocytosis in ovarian cancer cells. Cancer Res.

[19] Kabeya Y, Mizushima N, Ueno T, Yamamoto A, Kirisako T, Noda T, Kominami E, Ohsumi Y, Yoshimori T (2000). LC3, a mammalian homologue of yeast Apg8p, is localized in autophagosome membranes after processing. Embo J.

[20] Biederbick A, Kern HF, Elsasser HP (1995). Monodansylcadaverine (MDC) is a specific in vivo marker for autophagic vacuoles. Eur J Cell Biol.

[21] Noda T, Ohsumi Y (1998). Tor, a phosphatidylinositol kinase homologue, controls autophagy in yeast. J Biol Chem.

[22] Meijer AJ, Codogno P (2004). Regulation and role of autophagy in mammalian cells. Int J Biochem Cell Biol.

[23] Kissova I, Deffieu M, Samokhvalov V, Velours G, Bessoule JJ, Manon S, Camougrand N (2006). Lipid oxidation and autophagy in yeast. Free Radic Biol Med.

[24] Lee IH, Cao L, Mostoslavsky R, Lombard DB, Liu J, Bruns NE, Tsokos M, Alt FW, Finkel T (2008). A role for the NAD-dependent deacetylase Sirt1 in the regulation of autophagy. Proc Natl Acad Sci U S A.

[25] Yu R, Hebbar V, Kim DW, Mandlekar S, Pezzuto JM, Kong AN (2001). Resveratrol inhibits phorbol ester and UV-induced activator protein 1 activation by interfering with mitogenactivated protein kinase pathways. Mol Pharmacol.

[26] Matter WF, Brown RF, Vlahos CJ (1992). The inhibition of phosphatidylinositol 3-kinase by quercetin and analogs. Biochem Biophys Res Commun.

[27] Yu L, Alva A, Su H, Dutt P, Freundt E, Welsh S, Baehrecke EH, Lenardo MJ (2004). Regulation of an ATG7-beclin 1 program of autophagic cell death by caspase-8. Science.

[28] Scott RC, Schuldiner O, Neufeld TP (2004). Role and regulation of starvation-induced autophagy in the Drosophila fat body. Dev Cell.

[29] Mieulet V, Roceri M, Espeillac C, Sotiropoulos A, Ohanna M, Oorschot V, Klumperman J, Sandri M, Pende M (2007). S6 kinase inactivation impairs growth and translational target phosphorylation in muscle cells maintaining proper regulation of protein turnover. Am J Physiol Cell Physiol.

[30] Pende M, Um SH, Mieulet V, Sticker M, Goss VL, Mestan J, Mueller M, Fumagalli S, Kozma SC, Thomas G (2004). S6K1(-/-)/S6K2(-/-) mice exhibit perinatal lethality and rapamycinsensitive 5'-terminal oligopyrimidine mRNA translation and reveal a mitogen-activated protein kinase-dependent S6 kinase pathway. Mol Cell Biol.

[31] Kaeberlein M, McVey M, Guarente L (1999). The SIR2/3/4 complex and SIR2 alone promote longevity in Saccharomyces cerevisiae by two different mechanisms. Genes Dev.

[32] Rogina B, Helfand SL (2004). Sir2 mediates longevity in the fly through a pathway related to calorie restriction. Proc Natl Acad Sci U S A.

[33] Tissenbaum HA, Guarente L (2001). Increased dosage of a sir-2 gene extends lifespan in Caenorhabditis elegans. Nature.

[34] Billington RA, Genazzani AA, Travelli C, Condorelli F (2008). NAD depletion by FK866 induces autophagy. Autophagy.

[35] (1995). Blommaart EF, Luiken JJ, Blommaart PJ, van Woerkom GM, Meijer AJ. Phosphorylation of ribosomal protein S6 is inhibitory for autophagy in isolated rat hepatocytes. J Biol Chem.

[36] Lee SB, Kim S, Lee J, Park J, Lee G, Kim Y, Kim JM, Chung J (2007). ATG1, an autophagy regulator, inhibits cell growth by negatively regulating S6 kinase. EMBO Rep.

[37] Wei M, Fabrizio P, Hu J, Ge H, Cheng C, Li L, Longo VD (2008). Life span extension by calorie restriction depends on Rim15 and transcription factors downstream of Ras/PKA, Tor, and Sch9. PLoS Genet.

[38] Madia F, Gattazzo C, Wei M, Fabrizio P, Burhans WC, Weinberger M, Galbani A, Smith JR, Nguyen C, Huey S, Comai L, Longo VD (2008). Longevity mutation in SCH9 prevents recombination errors and premature genomic instability in a Werner/Bloom model system. J Cell Biol.

[39] Longo VD (2003). The Ras and Sch9 pathways regulate stress resistance and longevity. Exp Gerontol.

[40] Kapahi P, Zid BM, Harper T, Koslover D, Sapin V, Benzer S (2004). Regulation of lifespan in Drosophila by modulation of genes in the TOR signaling pathway. Curr Biol.

[41] Patel PH, Tamanoi F (2006). Increased Rheb-TOR signaling enhances sensitivity of the whole organism to oxidative stress. J Cell Sci.

[42] Um SH, Frigerio F, Watanabe M, Picard F, Joaquin M, Sticker M, Fumagalli S, Allegrini PR, Kozma SC, Auwerx J, Thomas G (2004). Absence of S6K1 protects against age- and diet-induced obesity while enhancing insulin sensitivity. Nature.

[43] Lamming DW, Wood JG, Sinclair DA (2004). Small molecules that regulate lifespan: evidence for xenohormesis. Mol Microbiol.

[44] Dasgupta B, Milbrandt J (2007). Resveratrol stimulates AMP kinase activity in neurons. Proc Natl Acad Sci U S A.

[45] Roux PP, Ballif BA, Anjum R, Gygi SP, Blenis J (2004). Tumor-promoting phorbol esters and activated Ras inactivate the tuberous sclerosis tumor suppressor complex via p90 ribosomal S6 kinase. Proc Natl Acad Sci U S A.

