# Supplementary material for: Inhibition of mammalian S6 kinase by resveratrol suppresses autophagy
Source: Aging (Albany NY). 2009 Jun 3;1(6):515–28. doi: 10.18632/aging.100056 (PMC2806030; doi:10.18632/aging.100056)
Supplement: Supplementary Table 1 [file aging-01-515-s001.doc]

**Supplemental** Table 1 – Kinase inhibition profile of resveratrol (20µM)

| No | Kinase | Activity (% of Control) |  | No | Kinase | Activity ( % of Control) |
| --- | --- | --- | --- | --- | --- | --- |
| 1 | Abl(h) | 105 ± 7 |  | 51 | MST2(h) | 73 ± 8 |
| 2 | Abl(T315I)(h) | 100 ± 2 |  | 52 | NEK2(h) | 102 ± 4 |
| 3 | ALK(h) | 39 ± 0 |  | 53 | NEK3(h) | 96 ± 8 |
| 4 | ALK4(h) | 110 ± 3 |  | 54 | NEK6(h) | 140 ± 16 |
| 5 | AMPK(r) | 39 ± 2 |  | 55 | NEK7(h) | 116 ± 2 |
| 6 | Aurora-A(h) | 65 ± 1 |  | 56 | NLK (h) | 30 ± 2 |
| 7 | CDK1/cyclinB(h) | 67 ± 3 |  | 57 | p70S6K(h) | 21 ± 1 |
| 8 | CDK2/cyclinA(h) | 80 ± 1 |  | 58 | PAK2(h) | 98 ± 4 |
| 9 | CDK2/cyclinE(h) | 80 ± 0 |  | 59 | PAK3(h) | 46 ± 0 |
| 10 | CDK3/cyclinE(h) | 75 ± 4 |  | 60 | PAK4(h) | 103 ± 4 |
| 11 | CDK5/p25(h) | 64 ± 4 |  | 61 | PAK6(h) | 103 ± 3 |
| 12 | CDK5/p35(h) | 77 ± 2 |  | 62 | PDGFRα(h) | 114 ± 2 |
| 13 | CDK6/cyclinD3(h) | 55 ± 2 |  | 63 | PDGFRß(h) | 119 ± 2 |
| 14 | CDK7/cyclinH/MAT1(h) | 95 ± 1 |  | 64 | PDK1(h) | 108 ± 7 |
| 15 | CDK9/cyclin T1(h) | 70 ± 3 |  | 65 | PI 3-Kinaseβ(h) | 107 ± 1 |
| 16 | CHK1(h) | 95 ± 2 |  | 66 | PI 3-Kinaseγ(h) | 101 ± 1 |
| 17 | CHK2(h) | 59 ± 6 |  | 67 | PI 3-Kinaseδ(h) | 106 ± 1 |
| 18 | cKit(h) | 68 ± 0 |  | 68 | Pim-1(h) | 6 ± 2 |
| 19 | CSK(h) | 94 ± 5 |  | 69 | Pim-2(h) | 14 ± 2 |
| 20 | cSRC(h) | 61 ± 1 |  | 70 | PKA(h) | 100 ± 0 |
| 21 | DAPK1(h) | 71 ± 3 |  | 71 | PKBα(h) | 94 ± 2 |
| 22 | DAPK2(h) | 67 ± 7 |  | 72 | PKBß(h) | 105 ± 3 |
| 23 | EGFR(h) | 90 ± 5 |  | 73 | PKBγ(h) | 89 ± 4 |
| 24 | EphB2(h) | 113 ± 1 |  | 74 | PKCα(h) | 114 ± 9 |
| 25 | FGFR1(h) | 58 ± 2 |  | 75 | PKCßI(h) | 103 ± 5 |
| 26 | Fgr(h) | 78 ± 5 |  | 76 | PKCγ(h) | 80 ± 8 |
| 27 | Flt1(h) | 52 ± 1 |  | 77 | PKCδ(h) | 92 ± 1 |
| 28 | Flt3(h) | 41 ± 1 |  | 78 | PKCθ(h) | 99 ± 5 |
| 29 | Fms(h) | 61 ± 2 |  | 79 | PKCζ(h) | 89 ± 0 |
| 30 | GSK3α(h) | 79 ± 4 |  | 80 | PKD2(h) | 88 ± 5 |
| 31 | GSK3ß(h) | 92 ± 0 |  | 81 | PKG1ß(h) | 56 ± 4 |
| 32 | IGF-1R(h) | 75 ± 0 |  | 82 | Plk3(h) | 95 ± 9 |
| 33 | IKKα(h) | 40 ± 1 |  | 83 | Ros(h) | 102 ± 4 |
| 34 | IKKß(h) | 116 ± 2 |  | 84 | Rsk1(h) | 70 ± 1 |
| 35 | IR(h) | 71 ± 5 |  | 85 | Rsk2(h) | 32 ± 0 |
| 36 | IRR(h) | 40 ± 0 |  | 86 | SAPK2a(h) | 100 ± 4 |
| 37 | IRAK1(h) | 97 ± 0 |  | 87 | SAPK2a(T106M)(h) | 102 ± 2 |
| 38 | JAK2(h) | 27 ± 5 |  | 88 | SAPK2b(h) | 100 ± 1 |
| 39 | JNK1α1(h) | 89 ± 5 |  | 89 | SAPK3(h) | 110 ± 5 |
| 40 | JNK2α2(h) | 110 ± 4 |  | 90 | SAPK4(h) | 92 ± 4 |
| 41 | JNK3(h) | 98 ± 3 |  | 91 | SGK(h) | 86 ± 13 |
| 42 | LIMK1(h) | 84 ± 4 |  | 92 | SGK2(h) | 70 ± 1 |
| 43 | LKB1(h) | 102 ± 3 |  | 93 | SIK(h) | 76 ± 1 |
| 44 | MAPK1(h) | 94 ± 13 |  | 94 | Snk(h) | 81 ± 3 |
| 45 | MAPK2(h) | 101 ± 10 |  | 95 | TAK1(h) | 105 ± 3 |
| 46 | MEK1(h) | 110 ± 5 |  | 96 | TBK1(h) | 62 ± 4 |
| 47 | Met(h) | 114 ± 5 |  | 97 | Tie2(h) | 94 ± 8 |
| 48 | MSK1(h) | 58 ± 1 |  | 98 | TrkA(h) | 31 ± 3 |
| 49 | MSK2(h) | 60 ± 1 |  | 99 | TrkB(h) | 98 ± 1 |
| 50 | MST1(h) | 75 ± 6 |  | 100 | ZAP-70(h) | 141 ± 2 |

Data set was performed in duplicate and represented as activity as a percent of DMSO control ± standard deviation. Numbers in the left column correspond to the values on the graph in Fig. 4.
