# Supplementary figures and images for: Inhibition of mammalian S6 kinase by resveratrol suppresses autophagy
Source: Aging (Albany NY). 2009 Jun 3;1(6):515–28. doi: 10.18632/aging.100056 (PMC2806030; doi:10.18632/aging.100056)

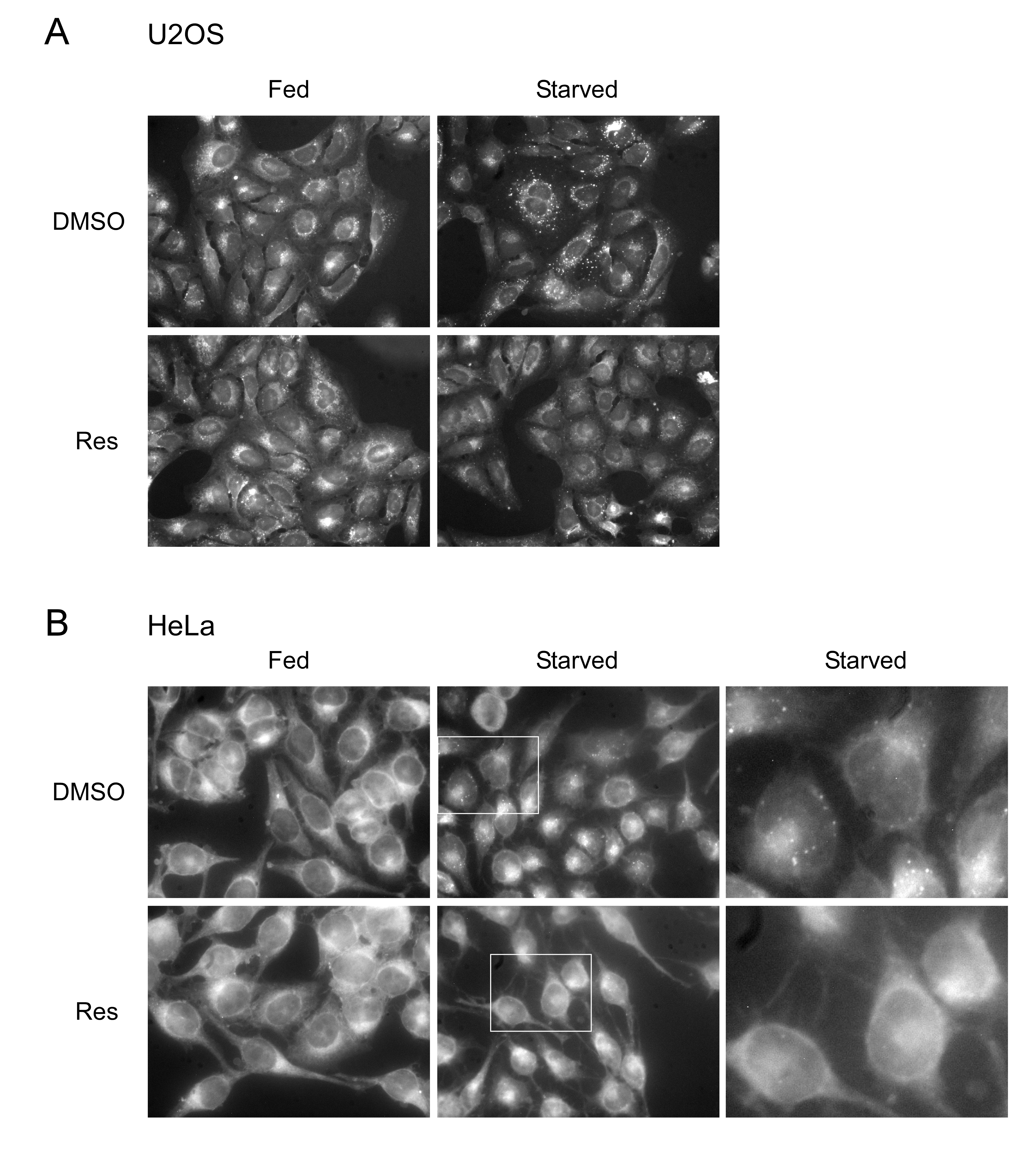

Supplement: Supplementary Figure 1 — (A) MDC staining of U2OS cells subjected to nutrient limitation (Starved) ± 50 μM resveratrol (Res) for 4 hours. (B) MDC staining of HeLa cells subjected to nutrient limitation (Starved) ± 50 μM resveratrol (Res) for 4 hours. An expansion of the area in the white box in the far right panels is displayed for clarity. [file aging-01-515-s001.tif]

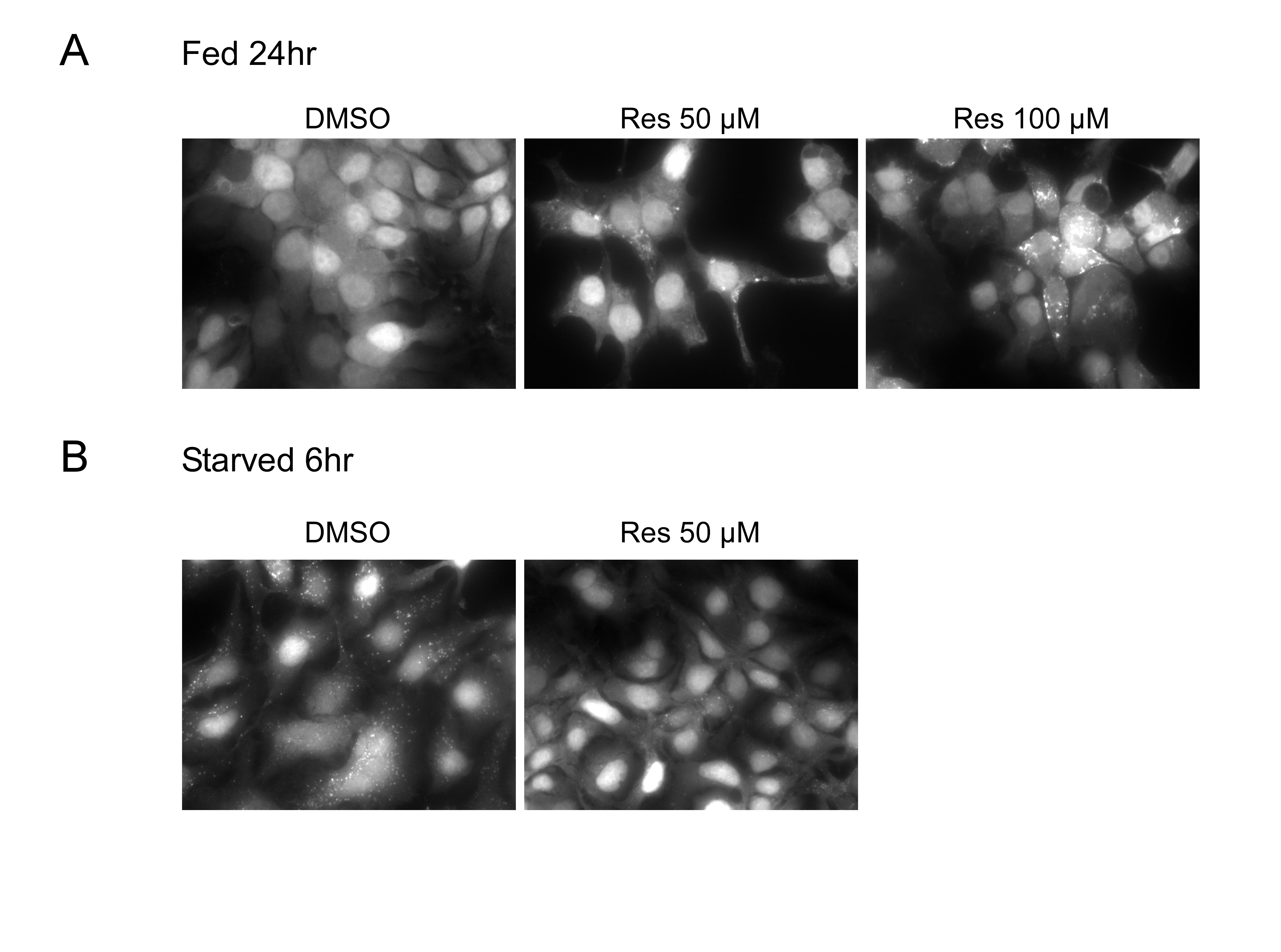

Supplement: Supplementary Figure 2 — (A) HEK293 GFP-LC3 expressing cells incubated in complete media plus serum were subjected to 50 or 100 μM resveratrol for 24 hours. (B) HEK293 GFP-LC3 cells were treated with EBSS (Starved) ±50 μM resveratrol (Res) for punctae comparison. [file aging-01-515-s002.tif]

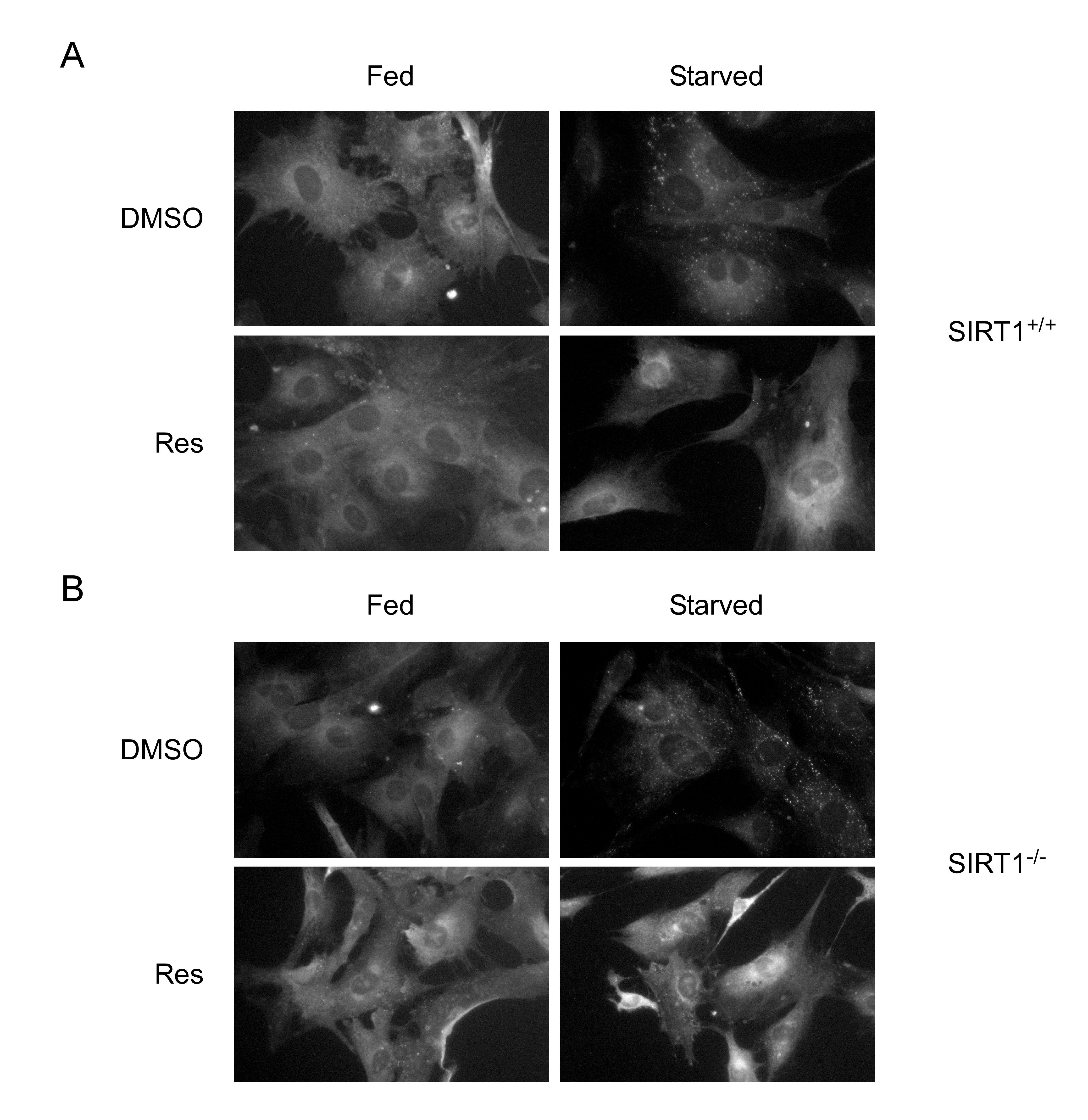

Supplement: Supplementary Figure 3 — (A) MDC staining of wild-type (SIRT1+/+) MEFs subjected to nutrient limitation (Starved) ± 200 μM resveratrol (Res) for 4 hours. (B) MDC staining of SIRT1-/- MEFs subjected to EBSS (Starved) ± 200 μM Res for 4 hours. [file aging-01-515-s003.tif]

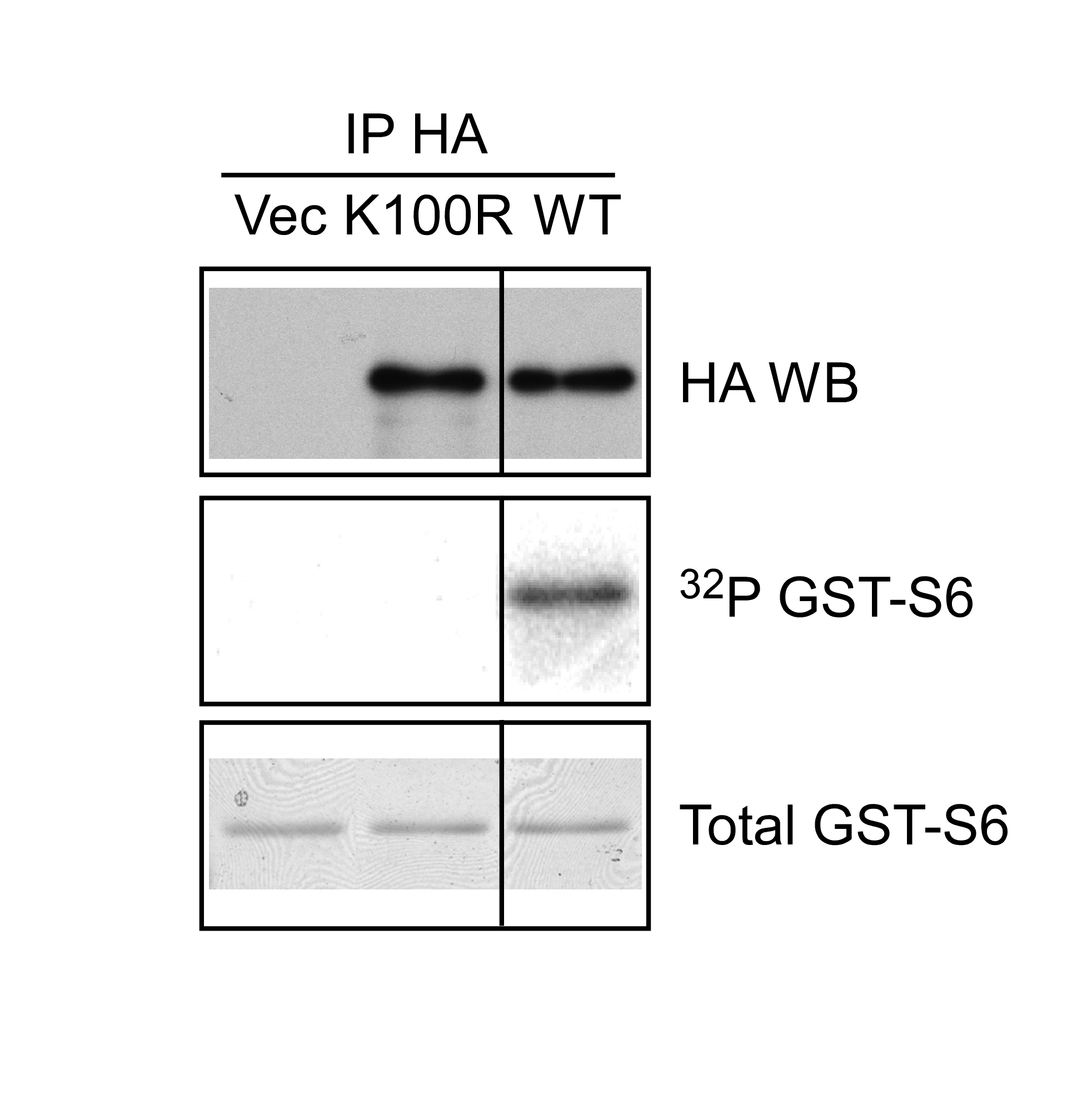

Supplement: Supplementary Figure 4 — HEK293 cells transfected with vector control (vec), kinase dead (K100R), or wild-type (WT) S6K1, were immunoprecipitated for HA-tagged S6K1, which was subsequently used to phosphorylate full-length GST-S6 ribosomal protein. Top panel is an HA western blot (WB). Middle panel is an autoradiogram indicating phosphorylated GST-S6. The bottom panel is a coomassie stained gel indicating the total GST-S6 in each lane. The black line indicates where the gel was cropped to include only the positive and negative controls for simplicity. [file aging-01-515-s004.tif]
